# Hypergraphs with edge-dependent vertex weights: *p*-Laplacians and spectral clustering

**DOI:** 10.3389/fdata.2023.1020173

**Published:** 2023-02-21

**Authors:** Yu Zhu, Santiago Segarra

**Affiliations:** Department of Electrical and Computer Engineering, Rice University, Houston, TX, United States

**Keywords:** submodular hypergraphs, *p*-Laplacian, spectral clustering, edge-dependent vertex weights, decomposable submodular function minimization

## Abstract

We study *p*-Laplacians and spectral clustering for a recently proposed hypergraph model that incorporates edge-dependent vertex weights (EDVW). These weights can reflect different importance of vertices within a hyperedge, thus conferring the hypergraph model higher expressivity and flexibility. By constructing submodular EDVW-based splitting functions, we convert hypergraphs with EDVW into submodular hypergraphs for which the spectral theory is better developed. In this way, existing concepts and theorems such as *p*-Laplacians and Cheeger inequalities proposed under the submodular hypergraph setting can be directly extended to hypergraphs with EDVW. For submodular hypergraphs with EDVW-based splitting functions, we propose an efficient algorithm to compute the eigenvector associated with the second smallest eigenvalue of the hypergraph 1-Laplacian. We then utilize this eigenvector to cluster the vertices, achieving higher clustering accuracy than traditional spectral clustering based on the 2-Laplacian. More broadly, the proposed algorithm works for all submodular hypergraphs that are graph reducible. Numerical experiments using real-world data demonstrate the effectiveness of combining spectral clustering based on the 1-Laplacian and EDVW.

## 1. Introduction

Spectral clustering makes use of eigenvalues and eigenvectors of graph Laplacians to group vertices in a graph. It is one of the most popular clustering methods due to its generality, efficiency, and strong theoretical basis. Standard graph Laplacians were first adopted to obtain relaxations of balanced graph cut criteria (Von Luxburg, 2007). Later, these were generalized to *p*-Laplacians, which are able to provide better approximations of the Cheeger constant (Amghibech, 2003; Bühler and Hein, 2009). Especially, the second smallest eigenvalue of the 1-Laplacian is identical to the Cheeger constant and the partition that achieves the optimal Cheeger cut can be obtained by thresholding the corresponding eigenvector (Szlam and Bresson, 2010).

Graphs are widely used to model pairwise interactions, but in many real-world applications the entities engage in higher-order relationships (Benson et al., 2016; Schaub et al., 2021). For instance, in co-authorship networks multiple authors may interact in writing an article together (Chitra and Raphael, 2019). In an e-commerce system, multiple customers can be associated if they once purchased the same product (Li et al., 2018). In text mining, multiple documents are related to each other if they contain the same keywords (Hayashi et al., 2020; Zhu et al., 2021). Such multi-way relations can be modeled by hypergraphs, where the notion of an edge is generalized to a hyperedge that can connect more than two vertices.

In graphs, there is only one way to cut an edge, thus, a scalar weight is enough to characterize the cut. But in hypergraphs, there may exist multiple ways to split a hyperedge. Consequently, a splitting function *w*_*e*_ is introduced for each hyperedge *e* in the hypergraph, assigning a cost to every possible cut of *e*. For any S⊆e, $w_{e}\left(S\right)$ indicates the penalty of partitioning *e* into S and e\S. In particular, when *w*_*e*_ is submodular for every hyperedge *e*, the corresponding model is termed as a submodular hypergraph which has desirable mathematical properties, making it convenient for theoretical analysis (Li and Milenkovic, 2017, 2018). A series of results in graph spectral theory including *p*-Laplacians, nodal domain theorems, and Cheeger inequalities have been generalized to submodular hypergraphs (Li and Milenkovic, 2018; Yoshida, 2019).

The choice of hyperedge splitting functions has a large practical effect on the hypergraph clustering performance. There are mainly two types of splitting functions in existing work. One is the so-called all-or-nothing splitting function in which an identical penalty is charged if the hyperedge is split regardless of how its vertices are separated (Hein et al., 2013). Another slightly more general type is the class of cardinality-based splitting functions where the splitting penalty depends only on the number of vertices placed on each side of the split (Veldt et al., 2020).

The limitation of existing splitting functions is that they treat all the vertices in a hyperedge equally while in practice these vertices may have different degrees of contribution to the hyperedge. Such information can be captured by edge-dependent vertex weights (EDVW): every vertex *v* is associated with a weight γ_*e*_(*v*) for each incident hyperedge *e* that reflects the contribution or importance of *v* to *e* (Chitra and Raphael, 2019). Going back to the aforementioned examples, EDVW can be used to model the author positions in a co-authored article, the quantity of a product bought by a customer, as well as the frequency of a word in a document.

Spectral theory on the hypergraph model with EDVW is much less developed than on submodular hypergraphs. Existing works studying hypergraphs with EDVW have only focused on random walk-based Laplacian matrices (Chitra and Raphael, 2019; Hayashi et al., 2020; Zhu et al., 2021), thus raising the question: *How to define non-linear* *p-Laplacians for the hypergraph model that incorporates EDVW?* Our basic idea for solving this problem is to convert a hypergraph with EDVW into a submodular hypergraph then *p*-Laplacians and related theorems developed for submodular hypergraphs can be directly leveraged. Based on our earlier work (Zhu et al., 2022), the model conversion can be achieved by defining submodular EDVW-based splitting functions in the form of $w_{e}\left(S\right)=g_{e}\left(\underset{v\text{∈}S}{\sum }\gamma_{e}\left(v\right)\right)$ where *g*_*e*_ is a concave function and the splitting penalty $w_{e}\left(S\right)$ is dependent only on the sum of EDVW in S. Moreover, hypergraphs with such splitting functions are proved to be graph reducible, meaning that there exists some graph sharing the same cut properties (Zhu and Segarra, 2022).

Since the 1-Laplacian provides the tightest approximation of the Cheeger constant, a follow-up question is: *How to apply* 1*-spectral clustering to submodular hypergraphs with EDVW-based splitting functions?* To this end, we develop an algorithm to compute the second eigenvector of the 1-Laplacian for EDVW-based submodular hypergraphs based on the inverse power method (IPM). The IPM was initially proposed for undirected graphs (Hein and Bühler, 2010), then generalized to submodular hypergraphs with cardinality-based splitting functions (Li and Milenkovic, 2018). A key to the success of IPM is an efficient solution to the inner-loop optimization problem in it. In this paper, we derive an equivalent definition of graph reducibility based on which we further propose an efficient solution to the inner problem that works for all graph reducible submodular hypergraphs including those with EDVW. The proposed solution can also be used to solve submodular function minimization (SFM) problems (Bach, 2013) when the submodular function can be represented as sums of concave functions applied to modular functions.

The major contributions of this paper can be summarized as follows:

(1) We present an equivalent definition of graph reducibility in terms of the Lovász extension of the cut function (see Theorem 1), which is helpful for understanding the relations between graph Laplacians and hypergraph Laplacians.
(2) We propose an algorithm to compute the eigenvector of the 1-Laplacian associated with the second smallest eigenvalue for all graph reducible submodular hypergraphs including those with EDVW-based splitting functions, and use the eigenvector for 1-spectral clustering.
(3) We validate the effectiveness of the proposed algorithm which leverages both of EDVW and 1-spectral clustering *via* numerical experiments on real-world datasets.

### 1.1. Paper outline

The rest of this paper is structured as follows. Preliminary mathematical concepts and submodular hypergraphs are reviewed in section 2. Section 3 introduces the hypergraph model with EDVW and shows how to convert it to graph reducible submodular hypergraphs by constructing EDVW-based splitting functions. The section also presents two equivalent definitions for graph reducibility. The proposed 1-spectral clustering algorithm is described in section 4. Section 5 presents experimental results. The relation between hypergraph Laplacians defined in different ways and the application of the proposed algorithm in SFM are discussed in section 6. Closing remarks are included in section 7.

### 1.2. Notation

For a vector **x** and a set S, ${\text{x}}_{S}$ denotes the vector formed by the entries of **x** indexed by S and $\text{x}\left(S\right)=\underset{v\in S}{\sum }x_{v}$. The operator $P_{a,b}\left(\text{x}\right)$ projects every entry of **x** onto the range [*a, b*]. Throughout the paper we assume that the considered hypergraphs are connected.

## 2. Preliminaries

### 2.1. Mathematical preliminaries

For a finite set V, a set function $F:2^{V}$ → ℝ is called submodular if $F\left(S_{1}\right)+F\left(S_{2}\right)\geq F\left(S_{1}\cup S_{2}\right)+F\left(S_{1}\cap S_{2}\right)$ for every $S_{1},S_{2}\subseteq V$. Considering a set function $F:2^{V}$ → ℝ such that *F*(∅) = 0 where V=[N]={1,2,⋯,N}, its Lovász extension *f*:ℝ^*N*^ → ℝ is defined as follows. For any **x** ∈ ℝ^*N*^, sort its entries in non-increasing order *x*_*i*_1__ ≥ *x*_*i*_2__ ≥ ⋯ ≥ *x*_*i*_*N*__, where (*i*_1_, *i*_2_, ⋯ , *i*_*N*_) is a permutation of (1, 2, ⋯ , *N*), and set


(1) $$\begin{array}{l} f\left(\text{x}\right)=\sum_{j=1}^{N-1}F\left(S_{j}\right)\left(x_{i_{j}}-x_{i_{j+1}}\right)+F\left(V\right)x_{i_{N}}, \end{array}$$

where $S_{j}=\left\{i_{1},\cdots \;,i_{j}\right\}$ for 1 ≤ *j* < *N*. A set function *F* is submodular if and only if its Lovász extension *f* is convex (Lovász, 1983). For any S⊆V, $F\left(S\right)=f\left(1_{S}\right)$ where $1_{S}$ is the indicator vector of S. If F(V)=0, *f*(*a* **x** + *b***1**) = *af* (**x**) for any *a* ∈ ℝ_≥0_, *b* ∈ ℝ. More properties of submodular functions can be found in Appendix A.

### 2.2. Submodular hypergraphs

Let $H=\left(V,E,\mu ,\left\{w_{e}\right\}\right)$ denote a submodular hypergraph (Li and Milenkovic, 2018) where V=[N] is the vertex set and E is the set of hyperedges. The function $\mu :V\to {\mathbb{R}}_{+}$ assigns positive weights to vertices. Each hyperedge e∈E is associated with a submodular splitting function $w_{e}:2^{e}\to {\mathbb{R}}_{\geq 0}$ that assigns non-negative penalties to every possible split of *e*. Moreover, *w*_*e*_ is required to satisfy *w*_*e*_(∅) = 0 and be symmetric so that $w_{e}\left(S\right)=w_{e}\left(e\backslash S\right)$ for any S⊆e. The domain of *w*_*e*_ can be extended from 2^*e*^ to $2^{V}$ by setting $w_{e}\left(S\right)=w_{e}\left(S\cap e\right)$ for any S⊆V, guaranteeing that the submodularity is maintained.

A cut is a partition of the vertex set V into two disjoint, non-empty subsets denoted by S and its complement V\S. The weight of the cut is defined as the sum of splitting penalties associated with each hyperedge (Li and Milenkovic, 2018; Veldt et al., 2020), i.e.,


(2) $$\begin{array}{l} {\text{cut}}_{H}\left(S\right)=\sum_{e\in E}w_{e}\left(S\right). \end{array}$$

The normalized Cheeger cut (NCC) is defined as


(3) $$\begin{array}{l} \text{NCC}\left(S\right)=\frac{{\text{cut}}_{H}\left(S\right)}{\min\left\{\text{vol}\left(S\right),\text{vol}\left(V\backslash S\right)\right\}} \end{array}$$

where $\text{vol}\left(S\right)=\underset{v\in S}{\sum }\mu \left(v\right)$ denotes the volume of S. The 2-way Cheeger constant is defined as the minimum NCC over all non-empty subsets of V except itself, i.e.,


(4) $$\begin{array}{l} h_{2}=\underset{\emptyset \text{⊂}S\text{⊂}V}{\min}\text{NCC}\left(S\right). \end{array}$$

The solution to (4) provides an optimal partitioning in the sense that we obtain two clusters which are balanced in terms of volume and loosely connected as captured by a small cut weight. In this paper, we adopt the minimization of NCC as our objective. There exist other clustering measures such as ratio cut, normalized cut and ratio Cheeger cut, which are closely related (Von Luxburg, 2007; Bühler and Hein, 2009).

Optimally solving (4) has been shown to be NP-hard for graphs (Wagner and Wagner, 1993), let alone hypergraphs. In spectral graph theory, Cheeger inequalities are derived to bound and approximate the Cheeger constant using graph *p*-Laplacians (Amghibech, 2003; Bühler and Hein, 2009; Szlam and Bresson, 2010; Chang, 2016; Chang et al., 2017; Tudisco and Hein, 2018). The results have been generalized to submodular hypergraphs (Li and Milenkovic, 2018; Yoshida, 2019). The *p*-Laplacian △_*p*_ of a submodular hypergraph is defined as an operator that, for all **x** ∈ ℝ^*N*^, induces


(5) $$\begin{array}{l} \langle \text{x},{△}_{p}\left(\text{x}\right)\rangle =\sum_{e\in E}\vartheta_{e}f_{e}{\left(\text{x}\right)}^{p}≜Q_{p}\left(\text{x}\right), \end{array}$$

where $\vartheta_{e}=\underset{S\subseteq e}{\max}w_{e}\left(S\right)$ and *f*_*e*_ is the Lovász extension of the normalized splitting function $\vartheta_{e}^{-1}w_{e}$. Notice that △_*p*_ can be alternatively defined in terms of the subdifferential of *f*_*e*_ (Li and Milenkovic, 2018), while we keep the definition (5) since it is more instrumental to our development. In particular, when *p* = 1, *Q*_1_(**x**) turns out to be the Lovász extension of the cut function defined in (2). It has been proved that the second smallest eigenvalue of the 1-Laplacian is identical to the Cheeger constant *h*_2_ (Li and Milenkovic, 2018).

## 3. EDVW-based submodular hypergraphs

### 3.1. The hypergraph model with EDVW

Let $H=\left(V,E,\mu ,\kappa ,\left\{\gamma_{e}\right\}\right)$ represent a hypergraph with EDVW (Chitra and Raphael, 2019) where V, E, and μ respectively denote the vertex set, the hyperedge set, and positive vertex weights. The function $\kappa :E\to {\mathbb{R}}_{+}$ assigns positive weights to hyperedges, and those weights can reflect the strength of connection. Each hyperedge e∈E is associated with a function $\gamma_{e}:V\to {\mathbb{R}}_{\geq 0}$ to assign edge-dependent vertex weights. For *v* ∈ *e*, γ_*e*_(*v*) is positive and measures the importance of the vertex *v* to the hyperedge *e*; for *v*∉*e*, γ_*e*_(*v*) is set to zero. For convenience, we define $\gamma_{e}\left(S\right)=\underset{v\in S}{\sum }\gamma_{e}\left(v\right)$.

The motivation of introducing EDVW is to enable the hypergraph model to describe the cases when the vertices in the same hyperedge contribute differently to this hyperedge. This information cannot be captured by hypergraphs adopting the all-or-nothing or cardinality-based splitting functions and is also hard to be directly described by submodular hypergraphs, but it can be conveniently represented *via* EDVW. For example, Chitra and Raphael (2019) studies the application of ranking authors in an academic citation network where authors and papers are respectively modeled as vertices and hyperedges. For a paper *e* and any author *v* of the paper, the corresponding EDVW γ_*e*_(*v*) is set to 2 if *v* is the first or last author, otherwise the weight is set to 1.

### 3.2. Building submodular hypergraphs from EDVW

In order to effectively handle EDVW while still leveraging existing results obtained for submodular hypergraphs, we consider the conversion from a hypergraph with EDVW $H=\left(V,E,\mu ,\kappa ,\left\{\gamma_{e}\right\}\right)$ to a submodular hypergraph $H=\left(V,E,\mu ,\left\{w_{e}\right\}\right)$. The basic idea is to keep V, E, and μ unchanged, and construct submodular splitting functions {*w*_*e*_} from EDVW {γ_*e*_} and hyperedge weights κ. After such a transformation, we can directly extend concepts such as *p*-Laplacians and related theorems proposed for the submodular hypergraph model (Li and Milenkovic, 2018) to the EDVW-based hypergraph model.

In our preliminary works (Zhu and Segarra, 2022; Zhu et al., 2022), we have proposed a class of submodular EDVW-based splitting functions in the following form


(6) $$\begin{array}{l} w_{e}\left(S\right)=h_{e}\left(\kappa \left(e\right)\right)\cdot g_{e}\left(\gamma_{e}\left(S\right)\right), \end{array}$$

where *h*_*e*_:ℝ_+_ → ℝ_+_ is an arbitrary function and *g*_*e*_:[0, γ_*e*_(*e*)] → ℝ_≥0_ is concave, symmetric with respect to γ_*e*_(*e*)/2, and satisfies *g*_*e*_(0) = 0. The resulting *w*_*e*_ is a valid splitting function that is non-negative, submodular, symmetric, and satisfies *w*_*e*_(∅) = 0. In practice, it is reasonable to select a non-decreasing function for *h*_*e*_ such as *h*_*e*_(*x*) = 1 and *h*_*e*_(*x*) = *x* since a larger hyperedge weight κ(*e*) is expected to lead to a larger splitting penalty for the same split of the hyperedge. Possible choices of *g*_*e*_ include *g*_*e*_(*x*) = *x*·(γ_*e*_(*e*) − *x*), *g*_*e*_(*x*) = min{*x*, γ_*e*_(*e*) − *x*}, and *g*_*e*_(*x*) = min{*x*, γ_*e*_(*e*) − *x, b*} where *b* is a positive parameter. Also notice that for trivial EDVW, namely γ_*e*_(*v*) = 1 for all *v* ∈ *e*, the splitting functions defined as (6) reduce to cardinality-based ones (Veldt et al., 2020).

### 3.3. Hypergraph-to-graph reductions

Submodular hypergraphs with splitting functions defined as (6) have a desirable property that they are graph reducible. In other words, they can project onto some graph which shares identical cut properties. Following Veldt et al. (2020), we consider the reduction of a hypergraph to a (possibly directed) graph with a potentially augmented vertex set. For a directed graph (digraph) G with vertex set $V_{G}$ and weighted adjacency matrix **A** whose entry *A*_*uv*_ denotes the weight of the directed edge pointing from *u* to *v*, its cut function is defined as


(7) $$\begin{array}{l} {\text{cut}}_{G}\left(S\right)=\sum_{u\in S,v\in V_{G}\backslash S}A_{uv}. \end{array}$$

In the following, we state a formal definition for graph reducibility in terms of cut weights, which is a variant of the definition in terms of hyperedge splitting functions stated in Veldt et al. (2020).

Definition 1. For a submodular hypergraph H with vertex set V, we say that its cut function ${\text{cut}}_{H}\left(S\right)$ is reducible to the cut function of a directed graph G with vertex set $V_{G}=V\cup \bar{V}$ where $\bar{V}$ is a set of auxiliary vertices if the following equality holds


(8) $$\begin{array}{l} {\text{cut}}_{H}\left(S\right)=\underset{T\subseteq \bar{V}}{\min}{\text{cut}}_{G}\left(S\cup T\right),\;\forall S\subseteq V. \end{array}$$

In the following theorem, we show an equivalent condition for graph reducibility regarding the Lovász extension of the cut function, which is beneficial for our later development of the 1-spectral clustering algorithm. The Lovász extension of the graph cut function can be written as


(9) $$\begin{array}{l} Q_{1}^{\left(g\right)}\left(\text{y}\right)=\sum_{u,v\in V_{G}}A_{uv}\max\left\{y_{u}-y_{v},0\right\} \end{array}$$

where **y** is a vector of length $V_{G}$.

Theorem 1. The equality presented in (8) is equivalent to


(10) $$\begin{array}{l} Q_{1}\left(\text{x}\right)=\underset{\bar{\text{x}}\in {\mathbb{R}}^{M}}{\min}Q_{1}^{\left(g\right)}\left(\text{y}\right),\;\forall \text{x}\in {\mathbb{R}}^{N} \end{array}$$

where *Q*_1_(**x**) and $Q_{1}^{\left(g\right)}\left(\text{y}\right)$ are respectively defined in (5) and (9), |V|=N, $|\bar{V}|=M$, and **y** ∈ ℝ^*N*+*M*^ is composed of ${\text{y}}_{V}=\text{x}$ and ${\text{y}}_{\bar{V}}=\bar{\text{x}}$.

Proof. The equivalence between (8) and (10) is proved in Appendix B.

It has been shown in Veldt et al. (2020) that all hypergraphs with submodular cardinality-based splitting functions are graph reducible. Our earlier work (Zhu and Segarra, 2022) has generalized the conclusion to hypergraphs with submodular EDVW-based splitting functions. In the following section, we propose a 1-spectral clustering algorithm for all submodular hypergraphs that are graph reducible including those EDVW-based ones, which are the focus of this paper.

## 4. Spectral clustering based on the 1-Laplacian

### 4.1. IPM-based 1-spectral clustering

We study spectral clustering algorithms for EDVW-based submodular hypergraphs leveraging the 1-Laplacian. As mentioned in section 2.2, for submodular hypergraphs the Cheeger constant *h*_2_ is equal to the second smallest eigenvalue λ_2_ of the 1-Laplacian △_1_. The corresponding optimal bipartition can be obtained by thresholding the eigenvector of △_1_ associated with λ_2_ (Li and Milenkovic, 2018). This eigenvector can be computed by minimizing


(11) $$\begin{array}{l} R_{1}(x)=\frac{Q_{1}(x)}{\min_{c\in \mathbb{R}}||x-c1|{|}_{1,\mu }}, \end{array}$$

where $\left||\text{x}|{|}_{1,\mu }=\underset{v\in V}{\sum }\mu \left(v\right)|x_{v}\right|$. Given the eigenvector **x**, a partitioning can be defined as $S=\left\{v\in V|x_{v}>t\right\}$ and its complement, where *t* is a threshold value. The optimal *t* can be determined as the one that minimizes the NCC in (3). The pipeline for the 1-spectral clustering algorithm is summarized in Algorithm 1.

**Algorithm 1:** 1-spectral clustering for hypergraphs with EDVW.

1: **Input:** hypergraph with EDVW $H=\left(V,E,\mu ,\kappa ,\left\{\gamma_{e}\right\}\right)$
2: Convert H to a submodular hypergraph by constructing submodular splitting functions based on (6)
3: Compute the second eigenvector of the hypergraph 1-Laplacian *via* the minimization of *R*_1_(**x**) in (11)
4: Threshold the obtained eigenvector to get the bipartition of V where we choose the threshold value as the one that minimizes the NCC in (3)

The minimization of *R*_1_(**x**) can be solved based on the inverse power method (Hein and Bühler, 2010; Li and Milenkovic, 2018), as outlined in Algorithm 2. Three functions are introduced: $\mu_{+}\left(\text{x}\right)=\underset{v\in V:x_{v}>0}{\sum }\mu \left(v\right)$, $\mu_{-}\left(\text{x}\right)=\underset{v\in V:x_{v}<0}{\sum }\mu \left(v\right)$ and $\mu_{0}\left(\text{x}\right)=\underset{v\in V:x_{v}=0}{\sum }\mu \left(v\right)$. Although this algorithm cannot guarantee convergence to the second eigenvector, the objective *R*_1_(**x**) is guaranteed to decrease and converge in the iterative process. Moreover, if we start from some point $\text{x}=1_{S}$ in Algorithm 2 where (S,V\S) is a given partition of V, then each step of the IPM-based method gives a partition that has a smaller (or at least equal) NCC value (see theorem 4.2 in Hein and Setzer, 2011). This implies that we can leverage the partition obtained *via* other methods as initialization. The algorithm was first proposed for the undirected graph setting (Hein and Bühler, 2010), then generalized to submodular hypergraphs with cardinality-based splitting functions (Li and Milenkovic, 2018). It is actually a special case of a more general class of minimization algorithms called RatioDCA and generalized RatioDCA proposed in Hein and Setzer (2011) and Tudisco et al. (2018) in order to handle more types of balanced graph cuts and modularity measures, respectively. The major difference between the graph setting and the hypergraph setting lies in how the inner-loop optimization problem (cf. line 5 in Algorithm 2) is solved.

**Algorithm 2:** IPM-based minimization of *R*_1_(**x**) (Hein and Bühler, 2010; Li and Milenkovic, 2018).

1: **Input:** submodular hypergraph $H=\left(V,E,\mu ,\left\{w_{e}\right\}\right)$ with *N* vertices, accuracy ϵ
2: **Initialization:** non-constant **x** ∈ ℝ^*N*^ subject to 0 ∈ argmin_*c*_||**x** − *c***1**||_1, μ_, λ ← *R*_1_(**x**)
3: **repeat**
4: $\forall v\;\in \;v,\;g_{v}\leftarrow \{\begin{array}{ll} \text{sgn}(x_{v})\cdot \mu (v), & \text{if }x_{v}\neq 0\\ \frac{\mu_{-}(x)-\mu_{+}(x)}{\mu_{0}(x)}\cdot \mu (v), & \text{if }x_{v}=0 \end{array}$
5: **x**←argmin_||**x**|| ≤ 1_*Q*_1_(**x**)−λ〈**x**, *g*〉 (**inner problem)**
6: *c*←argmin_*c*_||**x**−*c***1**||_1, μ_
7: **x** ← **x** − *c***1**
8: ${\text{λ}}^{\prime }\leftarrow \text{λ},\text{λ}\leftarrow R_{1}\left(\text{x}\right)$
9: **until** $\frac{\left|\text{λ}-{\text{λ}}^{\prime }\right|}{{\text{λ}}^{\prime }}<\epsilon$
10: **Output:** **x**

In Li and Milenkovic (2018), the authors solved the inner problem using a random coordinate descent method (Ene and Nguyen, 2015) together with a divide-and-conquer algorithm proposed in Jegelka et al. (2013). The computational complexity of the divide-and-conquer algorithm depends on the time of solving the problem $\underset{S\subseteq e}{\min}F\left(S\right)≜w_{e}\left(S\right)+\text{z}\left(S\right)$ for an arbitrary vector **z** ∈ ℝ^|*e*|^. For a cardinality-based splitting function, the solution to this problem can be found efficiently *via* a line search even when |*e*| is large. In the line search, we create a series of sets $S_{0}=\emptyset ,S_{1},\cdots \;,S_{\left|e\right|}$, where $S_{i}$ contains *i* vertices corresponding to the first *i* smallest entries in vector **z**. We compare their objective values $F\left(S_{i}\right)$ and identify the solution $S_{i^{*}}$ leading to the minimum objective value. However, such solution does not work for EDVW-based splitting functions. In the following section, we study the inner problem considering the EDVW-based case.

### 4.2. Solution to the inner problem

We propose efficient solutions to the inner problem for EDVW-based submodular hypergraphs leveraging the property that they are graph reducible. More generally speaking, the proposed solutions work for all graph reducible submodular hypergraphs. We first show that the inner problem is equivalent to another optimization problem (12) defined on the digraph obtained *via* the reduction.

Theorem 2. For any submodular hypergraph H with vertex set V, if it is reducible to a digraph G with vertex set $V_{G}=V\cup \bar{V}$ and edge set $E_{G}$, i.e., (8) or (10) holds, then the solution **x** to the inner problem in Algorithm 2 can be obtained (up to a scalar multiple) by setting $\text{x}={\text{y}}_{V}$ and $\text{y}\in {\mathbb{R}}^{\left|V_{G}\right|}$ is the solution to


(12) $$\begin{array}{l} \underset{||\text{y}|{|}_{2}\leq 1}{\min}Q_{1}^{\left(g\right)}\left(\text{y}\right)-\langle y,\tilde{g}\rangle \end{array}$$

where $\tilde{g}\in {\mathbb{R}}^{\left|V_{G}\right|}$ is a vector composed of ${\tilde{g}}_{V}=\text{λ}g$ and ${\tilde{g}}_{\bar{V}}=0$.

Proof. The proof can be found in Appendix C where we have used the equivalent definition for graph reducibility proposed in **Theorem 1**.

We present two ways for solving problem (12) in sections 4.2.1 and 4.2.2, respectively.

#### 4.2.1. Solving the inner problem *via* FISTA

Both of the original inner problem and its equivalent problem (12) are convex but non-smooth. Inspired by Hein and Bühler (2010), we derive a dual formulation (13) of problem (12). Compared to the primal problem, the objective function Ψ of the dual problem is smooth. Moreover, problem (13) can be efficiently solved using a fast iterative shrinkage-thresholding algorithm (FISTA) (Nesterov, 1983; Beck and Teboulle, 2009), which has a guaranteed convergence rate *O*(1/*k*^2^) where *k* is the number of iterations. FISTA requires an upper bound on the Lipschitz constant of the gradient of Ψ as the input, which is provided in (15). The steps of FISTA are summarized in Algorithm 3.

**Algorithm 3:** Solution of problem (13) with FISTA.

1: **Input:** A Lipschitz constant *L* of ∇Ψ
2: **Initialization:** ***α*** = ***β*** ∈ ℝ^*m*^, *t* = 1
3: **repeat**
4: ***α***′←***α***, $\alpha \leftarrow \beta -\frac{1}{L}\nabla \text{Ψ}\left(\beta \right)$
5: $\alpha \leftarrow P_{0,1}\left(\alpha \right)$
6: *t*′←*t*, $t\leftarrow \frac{1+\sqrt{1+4t^{2}}}{2}$
7: $\beta \leftarrow \alpha +\frac{t^{\prime }-1}{t}\left(\alpha -\alpha^{\prime }\right)$
8: **until** convergence or a predefined maximum number of iterations is reached
9: **Output:** ***α***

To make it clear, in the theorem below, the set ${\tilde{E}}_{G}$ (as well as the directed edge set $E_{G}$) contains *ordered* node pairs, meaning that (*u, v*) and (*v, u*) are different. The parameter *m* can be understood as the number of connected node pairs where the connection might be unidirectional or bidirectional.

Theorem 3. Define a set ${\tilde{E}}_{G}=\left\{\left(u,v\right)\in V_{G}\times V_{G}|\left(u,v\right)\in E_{G}\text{ or }\left(v,u\right)\in E_{G}\right\}$ and set $m=|{\tilde{E}}_{G}|/2$. The dual of problem (12) is


(13) $$\begin{array}{l} \underset{\begin{array}{c} \alpha \in {\left[0,1\right]}^{m}\\ \alpha_{uv}+\alpha_{vu}=1 \end{array}}{\min}\text{Ψ}\left(\alpha \right)≜||f_{A}\left(\alpha \right)-\tilde{g}|{|}_{2}^{2} \end{array}$$

where **α** is a vector of length *m* collecting all **α**_*uv*_ satisfying $\left(u,v\right)\in {\tilde{E}}_{G}$ and *u* < *v*. For the function $f_{A}:{\mathbb{R}}^{m}\to {\mathbb{R}}^{N+M}$, the *u*th element of *f*_*A*_(**α**) is


$$\begin{array}{l} {\left[f_{A}\left(\alpha \right)\right]}_{u}=\sum_{v|\left(u,v\right)\in {\tilde{E}}_{G}}A_{uv}\alpha_{uv}-A_{vu}\alpha_{vu}. \end{array}$$

The primal and dual variables are related as


(14) $$\begin{array}{l} \text{y}=-\frac{f_{A}\left(\alpha \right)-\tilde{g}}{||f_{A}\left(\alpha \right)-\tilde{g}|{|}_{2}}. \end{array}$$

The Lipschitz constant of the gradient of Ψ is upper bounded by


(15) $$\begin{array}{l} L=4\underset{u\in V_{G}}{\max}\sum_{v\in V_{G}}{\left(A_{uv}+A_{vu}\right)}^{2}. \end{array}$$

Proof. The proof is given in Appendix D.

In a nutshell, to solve the inner problem, we first compute the adjacency matrix of the digraph according to the graph reduction procedure proposed in Zhu and Segarra (2022). Then we solve the dual problem (13) using FISTA, and get the solution **y** to the primal problem (12) according to the relation between the primal and dual variables as shown in (14). Finally, we obtain the solution **x** to the original problem by taking the entries of **y** indexed by V.

#### 4.2.2. Solving the inner problem *via* PDHG

Problem (12) can also be solved using a primal-dual hybrid gradient (PDHG) algorithm (Chambolle and Pock, 2011, 2016a,b). Although both FISTA and PDHG ensure a quadratic convergence rate, it has been observed that PDHG can outperform FISTA in practice for clustering applications (Hein and Setzer, 2011).

PDHG is able to solve problems in the following form:


(16) $$\begin{array}{l} \underset{\text{y}\in {\mathbb{R}}^{N_{1}}}{\min}f_{1}\left(\text{y}\right)+f_{2}\left(\text{B}\text{y}\right), \end{array}$$

whose dual problem is


(17) $$\begin{array}{l} \underset{\text{z}\in {\mathbb{R}}^{N_{2}}}{\max}-f_{1}^{*}\left(-{\text{B}}^{⊤}\text{z}\right)-f_{2}^{*}\left(\text{z}\right), \end{array}$$

where $f_{1}:{\mathbb{R}}^{N_{1}}\to \left(-\infty ,+\infty \right]$ and $f_{2}:{\mathbb{R}}^{N_{2}}\to \left(-\infty ,+\infty \right]$ are proper, convex, lower semicontinuous functions, $\text{B}:{\mathbb{R}}^{N_{1}}\to {\mathbb{R}}^{N_{2}}$ is a bounded linear operator, and $f_{1}^{*}$ and $f_{2}^{*}$ are the corresponding conjugate functions of *f*_1_ and *f*_2_ (Boyd et al., 2004).

The solution to problem (12) can be obtained *via* normalizing the solution to problem (18) below.


(18) $$\begin{array}{l} \underset{\text{y}\in {\mathbb{R}}^{\left|V_{G}\right|}}{\min}Q_{1}^{\left(g\right)}\left(\text{y}\right)+\frac{1}{2}||\text{y}-\tilde{g}|{|}_{2}^{2}. \end{array}$$

We can fit (18) into the form (16) by setting $N_{1}=|V_{G}|$, $N_{2}=|E_{G}|$, $f_{1}\left(\text{y}\right)=\frac{1}{2}||\text{y}-\tilde{g}|{|}_{2}^{2}$, $f_{2}\left(\text{z}\right)=\overset{N_{2}}{\underset{i=1}{\sum }}\max\left\{z_{i},0\right\}$, and **B** is a $\left|E_{G}|\times |V_{G}\right|$ matrix. For the row of **B** corresponding to edge *u* → *v*, the *u*th and *v*th elements in the row are respectively equal to *A*_*uv*_ and −*A*_*uv*_, and the other elements are zero. We can show that $f_{1}^{*}\left(\text{y}\right)=\frac{1}{2}||\text{y}|{|}_{2}^{2}+\langle \text{y},\tilde{\text{g}}\rangle$, $f_{2}^{*}\left(\text{z}\right)=0$ with the domain that 0 ≤ *z*_*i*_ ≤ 1 for any 0 ≤ *i* ≤ *N*_2_. Since *f*_1_ is 1-strongly convex, we can leverage an accelerated variant of the PDHG algorithm. The algorithm tailored for problem (18) is given in Algorithm 4 (cf. algorithm 8 in Chambolle and Pock, 2016a).

**Algorithm 4:** Solution of problem (18) with accelerated PDHG.

1: **Initialization:** $\tau \sigma \leq \frac{1}{||\text{B}|{|}_{2}^{2}}$, $\text{y}=\bar{\text{y}}\in {\mathbb{R}}^{\left|V_{G}\right|}$, $\text{z}\in {\mathbb{R}}^{\left|E_{G}\right|}$
2: **repeat**
3: $\text{z}\leftarrow P_{0,1}\left(\text{z}+\sigma \text{B}\bar{\text{y}}\right)$
4: **y**′←**y**, $\text{y}\leftarrow \frac{1}{1+\tau }\left(\text{y}-\tau \left({\text{B}}^{⊤}\text{z}-\tilde{g}\right)\right)$
5: $\theta \leftarrow \frac{1}{\sqrt{1+\tau }}$, τ←θτ, $\sigma \leftarrow \frac{\sigma }{\theta }$
6: $\bar{\text{y}}\leftarrow \text{y}+\theta \left(\text{y}-{\text{y}}^{\prime }\right)$
7: **until** convergence or a predefined maximum number of iterations is reached
8: **Output:** **y**

### 4.3. A special case: Reduction to an undirected graph

If the submodular hypergraph is reducible to an undirected graph (e.g., see theorem 3.3 in Zhu and Segarra, 2022), then there exists another dual formulation of problem (12) that bears a similar form to (13) while the gradient of its objective has a smaller Lipschitz constant (cf. lemma 4.3 in Hein and Bühler, 2010). In fact, for this case, the hypergraph 1-spectral clustering can be implemented *via* its graph counterpart.

Following **Theorem 1**, we further define a vertex weight function $\mu_{G}$ for the graph G such that $\mu_{G}\left(v\right)=\mu \left(v\right)$ for v∈V and $\mu_{G}\left(v\right)=0$ for each auxiliary vertex $v\in \bar{V}$. Then it follows from (10) that $R_{1}\left(\text{x}\right)=\underset{\bar{\text{x}}\in {\mathbb{R}}^{M}}{\min}R_{1}^{\left(g\right)}\left(\text{y}\right)$ for any **x** ∈ ℝ^*N*^ where $R_{1}^{\left(g\right)}\left(\text{y}\right)=\frac{Q_{1}^{\left(g\right)}\left(\text{y}\right)}{\underset{c\in \mathbb{R}}{\min}||\text{y}-c1|{|}_{1,\mu_{G}}}$ and **y** is composed of ${\text{y}}_{V}=\text{x}$ and ${\text{y}}_{\bar{V}}=\bar{\text{x}}$. Minimizing both sides of the equation over **x** ∈ ℝ^*N*^ leads to the same minimum NCC value. When G is undirected, the second eigenvector of the graph 1-Laplacian can be computed by minimizing $R_{1}^{\left(g\right)}\left(\text{y}\right)$ and then the second eigenvector of the hypergraph 1-Laplacian can be computed as $\text{x}={\text{y}}_{V}$. This can also be understood in terms of the cut function. Following (8), it is easy to show that the Cheeger constant of the graph G is identical to that of the hypergraph H. This coincides with theorem 3.3 in (Liu et al., 2021), which further proves that if S is the set in G leading to the minimum NCC for the vertex weight function $\mu_{G}$, then S∩V is the minimum NCC set in H.

## 5. Experiments

We evaluate the performance of the proposed 1-spectral clustering algorithm for EDVW-based submodular hypergraphs (termed as EDVW-based hereafter) by focusing on the bipartition case. In particular, for hyperedge splitting functions as in (6), we select *h*_*e*_(*x*) = *x* and *g*_*e*_(*x*) = min{*x*, *γ*(*e*) − *x*, *βγ*(*e*)} for every hyperedge e∈E, namely


(19) $$\begin{array}{l} w_{e}\left(S\right)=\kappa \left(e\right)\cdot \min\left\{\gamma_{e}\left(S\right),\gamma_{e}\left(e\right)-\gamma_{e}\left(S\right),\beta \gamma_{e}\left(e\right)\right\} \end{array}$$

where β is tunable. Notice that we reuse the symbols **α** and **β** in this section, which are different from and not related to **α** and **β** used when describing FISTA. A hypergraph with splitting functions as (19) can be reduced to a digraph that consists of |V|+2|E| vertices and $\left|E|+2\underset{e\in E}{\sum }|e\right|$ edges. More precisely, we first project each hyperedge *e* onto a small graph which contains |*e*| + 2 vertices including two auxiliary vertices denoted by *e*′ and *e*″. For every *v* ∈ *e*, there are two directed edges respectively from *v* to *e*′ and from *e*″ to *v*, both of weight κ(*e*)*γ*_*e*_(*v*). There is also a directed edge of weight *βκ*(*e*)*γ*_*e*_(*e*) from *e*′ to *e*″. Then we concatenate these small graphs for all hyperedges together to form the final graph.

### 5.1. Datasets

We consider three widely used real-world datasets.

*Reuters Corpus Volume 1 (RCV1):* This dataset is a collection of manually categorized newswire stories (Lewis et al., 2004). We consider two categories C14 and C23. A few short documents containing less than 20 words are ignored. We select the 100 most frequent words in the corpus after removing stop words and words appearing in >10 and < 0.2% of the documents. We then remove documents containing < 5 selected words, leaving us with 7, 446 documents. A document (vertex) belongs to a word (hyperedge) if the word appears in the document. The edge-dependent vertex weights are taken as the corresponding tf-idf (term frequency-inverse document frequency) values (Leskovec et al., 2020) to the power of α, where α is a tunable parameter.

20 *Newsgroups* ^1^*:* This is also a text dataset. For our two-partition case, we consider the documents in categories “rec.autos" and “rec.sport.hockey." We preprocess the dataset following the same procedure as used in RCV1 above. We finally construct a hypergraph of 1, 389 vertices and 100 hyperedges.

*Covtype:* This dataset contains patches of forest that are in different cover types. We consider two cover types (labeled as 4 and 5) and all numerical features. Each numerical feature is first quantized into 20 bins of equal range and then mapped to hyperedges. The resulting hypergraph has 12, 240 vertices and 196 hyperedges. For each hyperedge (bin), we compute the distance between each feature value in this bin and their median, and then normalize these distances to the range [0, 1]. The edge-dependent vertex weights are computed as exp(−α·distance). Under this setting, larger edge-dependent vertex weights are assigned to vertices whose feature values are close to the typical feature value in the corresponding bin.

Following Hayashi et al. (2020), for all datasets we set the hyperedge weight κ(*e*) to the standard deviation of the EDVW *γ*_*e*_(*v*) for all v∈V. Following Li and Milenkovic (2018), we set the vertex weight μ(*v*) to the vertex degree defined in the submodular hypergraph model, i.e., $\mu \left(v\right)=\underset{e∋v}{\sum }\vartheta_{e}$.

### 5.2. Baselines

We compare the proposed approach with three baseline methods.

*Random walk-based:* The paper Hayashi et al. (2020) defines a hypergraph Laplacian matrix based on random walks with EDVW. We compute the second eigenvector of the normalized hypergraph Laplacian [cf. (6) in Hayashi et al., 2020] and then threshold it to get the partitioning.

*Cardinality-based:* In the description of the datasets above, when α = 0, we get the trivial EDVW and the splitting functions reduce to cardinality-based ones.

*All-or-nothing:* For the adopted splitting functions (19), they reduce to the all-or-nothing case if β is small enough [i.e., $\beta \leq \underset{v\in e}{\min}\gamma_{e}\left(v\right)$].

For the proposed method, in Algorithm 2 we adopt the eigenvector obtained in the random walk-based method described above as the starting point. We solve the inner problem using PDHG presented in Algorithm 4. Moreover, in Algorithm 4 we initialize $\tau =\sigma =\frac{0.9}{||\text{B}|{|}_{2}}$ and **z** = **0**.

### 5.3. Results

**Figure 1:**
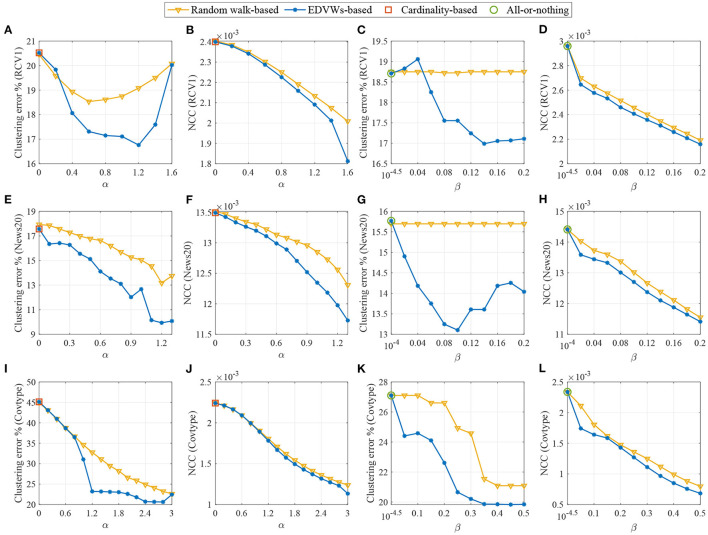
Clustering performance for three real-world datasets displayed in pairs of figures depicting the clustering error and the NCC value as a function of the parameters α or β. For RCV1, we fix β = 0.2 in **(A, B)** and α = 1 in **(C, D)**. For 20 Newsgroups, we fix β = 0.1 in **(E, F)** and α = 0.8 in **(G, H)**. For Covtype, we fix β = 0.2 in **(I, J)** and α = 2 in **(K, L)**. A proper choice of α and β helps significantly decrease the clustering error compared with existing methods. The performance improvement may benefit from both the use of EDVW and 1-Laplacian.

The results are shown in Figure 1. We present both the clustering error and the NCC where the clustering error is computed as the fraction of incorrectly clustered samples. For the RCV1 dataset (A–D), we fix β = 0.2 to observe the influence of α in (A, B) and fix α = 1 to test β in (C, D). For the 20 Newsgroups dataset (E–H), we fix β = 0.1 in (E, F) and α = 0.8 in (G, H) to observe the effects of α and β, respectively. For the Covtype dataset (I–L), we fix β = 0.2 in (I, J) and α = 2 in (K, L). We can see that for all the considered datasets, when edge-dependent vertex weights are ignored (including α = 0 for cardinality-based splitting functions and β close to zero for the all-or-nothing splitting function), the clustering performance is severely deteriorated, validating that it is necessary to incorporate EDVW for extra modeling flexibility. It can also be observed that, when appropriate values for α and β are selected (such as α = 1.2, β = 0.2 for RCV1, α = 1.2, β = 0.1 for 20 Newsgroups and α = 2.4, β = 0.2 for Covtype), the proposed method performs much better than the random walk-based method which depends on the classical Laplacian. This highlights the value of using the non-linear 1-Laplacian in spectral clustering. To summarize, both the use of EDVW and 1-Laplacian are beneficial for improving the spectral clustering performance.

## 6. Related work

We discuss the related works in this section. We will show the relationship between hypergraph Laplacians introduced *via* random walks and those defined based on submodular splitting functions. We will also show how the proposed solution to the inner problem contributes to SFM.

### 6.1. Random walk-based Laplacians

Here we expand the discussion about the relation between hypergraph Laplacians based on random walks with EDVW and those studied in this paper considering submodular EDVW-based splitting functions.

Considering the hypergraph model with EDVW (cf. section 3.1), the random walk incorporating EDVW is defined as follows (Chitra and Raphael, 2019). Starting at some vertex *u*, the walker selects a hyperedge *e* containing *u* with probability proportional to κ(*e*), then moves to vertex *v* contained in *e* with probability proportional to *γ*_*e*_(*v*). In this process, the probability of moving from *u* to *v* *via* *e* is


(20) $$\begin{array}{l} P_{u\to e\to v}=\left\{ \begin{array}{l} \frac{\kappa (e)}{\sum_{e^{\prime }∋u}\kappa (e^{\prime })}\cdot \frac{\gamma_{e}(v)}{\gamma_{e}(e)},\;u,v\in e,\\ 0,\text{ else}. \end{array}\right. \end{array}$$

Then one can define a transition matrix **P** whose (*u, v*)th entry denotes the transition probability from *u* to *v* and is computed as $P_{uv}=\underset{e\in E}{\sum }P_{u\to e\to v}$. When the hypergraph is connected, the random walk converges to a unique stationary distribution **π** which is the all-positive dominant left eigenvector of **P** scaled to satisfy ||**π**||_1_ = 1. In Chitra and Raphael (2019) and Hayashi et al. (2020), hypergraph Laplacians based on such random walks are proposed and they are actually equal to combinatorial and normalized graph Laplacian matrices (i.e., graph 2-Laplacians) of an undirected graph which is defined on the same vertex set as the hypergraph and has the following adjacency matrix


(21) $$\begin{array}{l} \text{A}=\frac{\Phi \text{P}+\text{P}\Phi }{2} \end{array}$$

where **Φ** is a diagonal matrix whose (*v, v*)th entry is π_*v*_.

Consider a hypergraph H with submodular splitting functions in the following form for each of its hyperedges:


(22) $$\begin{array}{l} w_{e}\left(S\right)=\sum_{u\in S,v\in e\backslash S}\frac{\pi_{u}P_{u\to e\to v}+\pi_{v}P_{v\to e\to u}}{2},\;\forall S\subseteq e. \end{array}$$

It is easy to show that H is reducible to the graph G defined by the adjacency matrix (21) since they have the same cut function as ${\text{cut}}_{H}\left(S\right)={\text{cut}}_{G}\left(S\right)=\sum_{u\in S,v\in V\backslash S}\frac{\pi_{u}P_{uv}+\pi_{v}P_{vu}}{2}$. Notice that there are no auxiliary vertices introduced in the graph reduction. Following **Theorem 1**, we have $Q_{1}\left(\text{x}\right)=Q_{1}^{\left(g\right)}\left(\text{x}\right)$, implying that the hypergraph 1-Laplacian of H is identical to the graph 1-Laplacian of G if we assume that they share the same vertex weight function μ.

### 6.2. Decomposable submodular function minimization

In the inner problem of Algorithm 2, if the norm of **x** is ||**x**||_∞_, the problem is equivalent to the following SFM problem


(23) $$\begin{array}{l} \underset{S\subseteq V}{\min}\sum_{e\in E}w_{e}\left(S\right)-\lambda g\left(S\right), \end{array}$$

where the primal and dual variables are related as *x*_*v*_ = 1 if v∈S and *x*_*v*_ = −1 if v∉S (Li and Milenkovic, 2018). Hence, the proposed solution to the inner problem can also be used to solve problems in the form of (23) when each function *w*_*e*_ can be represented as a concave function applied to a modular (additive) function [cf. (6)].

## 7. Conclusion

We presented an equivalent definition of graph reducibility based on which we further proposed a 1-spectral clustering algorithm for submodular hypergraphs that are graph reducible, especially for those with EDVW-based splitting functions. Through experiments on real-world datasets, we showcased the value of combining the hypergraph 1-Laplacian and EDVW. Future research directions include: (1) Developing computation methods for the hypergraph 1-Laplacian's eigenvectors which can work efficiently for all submodular splitting functions, (2) Designing multi-way partitioning algorithms based on non-linear Laplacians (Bühler and Hein, 2009), and (3) Exploring applications of *p*-Laplacians for different values of *p* (Fu et al., 2022).

## Data availability statement

The original contributions presented in the study are included in the article/Supplementary material, further inquiries can be directed to the corresponding author.

## Author contributions

YZ developed the methodology, conducted the experiments, and wrote the initial manuscript. SS supervised the study, contributed to the discussions, and edited the manuscript. All authors read and approved the final manuscript.

## Footnotes

^1^ http://qwone.com/~jason/20Newsgroups/
