# Supplementary material for: Hypergraphs with edge-dependent vertex weights: p-Laplacians and spectral clustering
Source: Front Big Data. 2023 Feb 21;6:1020173. doi: 10.3389/fdata.2023.1020173 (PMC9989290; doi:10.3389/fdata.2023.1020173)
Supplement: Supplementary file 1 [file Data_Sheet_1.pdf]

APPENDIX

A PRELIMINARY PROOFS

In this section, we present some properties of submodular functions which are useful in the derivations of the proposed theorems.

LEMMA 1 (Proposition 3.7 in (Bach, 2013)). *Let $F : 2^{\mathcal{V}} \to \mathbb{R}$ be a submodular function such that $\mathcal{V} = [N]$ and $F(\emptyset) = 0$, and denote its Lovász extension by f, then*

$$\min_{\mathcal{S}\subseteq\mathcal{V}} F(\mathcal{S}) = \min_{\mathbf{x}\in\{0,1\}^N} f(\mathbf{x}) = \min_{\mathbf{x}\in[0,1]^N} f(\mathbf{x}). \tag{24}$$

Moreover, the set of minimizers of $f(\mathbf{x})$ on $[0,1]^N$ is the convex hull of minimizers of $f(\mathbf{x})$ on $\{0,1\}^N$.

In the following, we use G to denote a submodular function defined on the power set of $\mathcal{V} \cup \bar{\mathcal{V}}$ where $|\mathcal{V}| = N$, $|\bar{\mathcal{V}}| = M$ and $\mathcal{V} \cap \bar{\mathcal{V}} = \emptyset$. We assume that $G(\emptyset) = 0$ and denote its Lovász extension by $g(\mathbf{y}) = g(\mathbf{x}, \bar{\mathbf{x}})$ for any $\mathbf{y} = [\mathbf{x}^\top, \bar{\mathbf{x}}^\top]^\top$ where $\mathbf{x} \in \mathbb{R}^N$ and $\bar{\mathbf{x}} \in \mathbb{R}^M$ respectively correspond to $\mathcal{V}$ and $\bar{\mathcal{V}}$.

LEMMA 2 (Proposition B.3 in (Bach, 2013)). *Define a set function $F : 2^{\mathcal{V}} \to \mathbb{R}$ based on G as*

$$F(\mathcal{S}) = \min_{\mathcal{T}\subseteq\bar{\mathcal{V}}} G(\mathcal{S} \cup \mathcal{T}) - \min_{\mathcal{T}\subseteq\bar{\mathcal{V}}} G(\mathcal{T}) \tag{25}$$

for any $\mathcal{S} \subseteq \mathcal{V}$. The function F is submodular and satisfies $F(\emptyset) = 0$. If $\min_{\mathcal{T}\subseteq\bar{\mathcal{V}}} G(\mathcal{T}) = 0$, the Lovász extension of F is such that for all $\mathbf{x} \in \mathbb{R}^N_{\geq 0}$,

$$f(\mathbf{x}) = \min_{\bar{\mathbf{x}}\in\mathbb{R}^M_{\geq 0}} g(\mathbf{x}, \bar{\mathbf{x}}). \tag{26}$$

LEMMA 3. *Further assume that the submodular function G is non-negative and satisfies $G(\mathcal{V} \cup \bar{\mathcal{V}}) = 0$. Given some $\mathbf{x} \in \mathbb{R}^N$ whose minimum element and maximum element are respectively a and b, the inequality*

$$g(\mathbf{x}, \bar{\mathbf{x}}) \geq g(\mathbf{x}, \mathcal{P}_{a,b}(\bar{\mathbf{x}})) \tag{27}$$

holds for any $\bar{\mathbf{x}} \in \mathbb{R}^M$. This implies that

$$\min_{\bar{\mathbf{x}}\in\mathbb{R}^M} g(\mathbf{x}, \bar{\mathbf{x}}) = \min_{\bar{\mathbf{x}}\in[a',b']^M} g(\mathbf{x}, \bar{\mathbf{x}}) = \min_{\bar{\mathbf{x}}\in[a,b]^M} g(\mathbf{x}, \bar{\mathbf{x}}) \tag{28}$$

for any $a' < a$ and $b' > b$.

PROOF. The proof follows immediately from the definition of the Lovász extension and thus is omitted here.

LEMMA 4. *Following Lemma 2, if G further satisfies the conditions listed in Lemma 3, then the Lovász extension of F is such that for all $\mathbf{x} \in \mathbb{R}^N$,*

$$f(\mathbf{x}) = \min_{\bar{\mathbf{x}}\in\mathbb{R}^M} g(\mathbf{x}, \bar{\mathbf{x}}). \tag{29}$$

PROOF. Given some $\mathbf{x} \in \mathbb{R}^N$ whose minimum element is a, we have

$$f(\mathbf{x}) = f(\mathbf{x} - a\mathbf{1}) \overset{(a)}{=} \min_{\bar{\mathbf{x}} \in \mathbb{R}^M_{\geq 0}} g(\mathbf{x} - a\mathbf{1}, \bar{\mathbf{x}}) = \min_{\bar{\mathbf{x}} \in \mathbb{R}^M_{\geq 0}} g(\mathbf{x}, \bar{\mathbf{x}} + a\mathbf{1}) = \min_{\bar{\mathbf{x}} \in [a,\infty)^M} g(\mathbf{x}, \bar{\mathbf{x}}) \overset{(b)}{=} \min_{\bar{\mathbf{x}} \in \mathbb{R}^M} g(\mathbf{x}, \bar{\mathbf{x}})$$

where (a) followed (26) and (b) followed Lemma 3.

LEMMA 5. *Define a set function $F : 2^{\bar{\mathcal{V}}} \to \mathbb{R}$ based on G as*

$$F(\mathcal{T}) = G(\mathcal{S} \cup \mathcal{T}) - G(\mathcal{S}) \tag{30}$$

for any $\mathcal{T} \subseteq \bar{\mathcal{V}}$. The function F is submodular and satisfies $F(\emptyset) = 0$. The Lovász extension of F is such that for all $\bar{\mathbf{x}} \in [0, 1]^M$,

$$f(\bar{\mathbf{x}}) = g(\mathbf{1}_{\mathcal{S}}, \bar{\mathbf{x}}) - G(\mathcal{S}). \tag{31}$$

PROOF. For any $\mathcal{T}_1, \mathcal{T}_2 \subseteq \bar{\mathcal{V}}$, one has

$$\begin{aligned}
&F(\mathcal{T}_1 \cup \mathcal{T}_2) + F(\mathcal{T}_1 \cap \mathcal{T}_2) \\
\overset{(a)}{=}&G(\mathcal{S} \cup [\mathcal{T}_1 \cup \mathcal{T}_2]) + G(\mathcal{S} \cup [\mathcal{T}_1 \cap \mathcal{T}_2]) - 2G(\mathcal{S}) \\
\overset{(b)}{=}&G([\mathcal{S} \cup \mathcal{T}_1] \cup [\mathcal{S} \cup \mathcal{T}_2]) + G([\mathcal{S} \cup \mathcal{T}_1] \cap [\mathcal{S} \cup \mathcal{T}_2]) - 2G(\mathcal{S}) \\
\overset{(c)}{\leq}&G(\mathcal{S} \cup \mathcal{T}_1) + G(\mathcal{S} \cup \mathcal{T}_2) - 2G(\mathcal{S}) \\
\overset{(d)}{=}&F(\mathcal{T}_1) + F(\mathcal{T}_2)
\end{aligned}$$

where (a) and (d) followed (30), (b) leveraged properties of set operations, and (c) was from the submodularity of the function G. Hence, the function F is proved to be submodular.

For any $\bar{\mathbf{x}} \in [0, 1]^M$, sort its entries in non-increasing order $1 \geq \bar{x}_{i_1} \geq \bar{x}_{i_2} \geq \cdots \geq \bar{x}_{i_M} \geq 0$ and define $\mathcal{T}_j = \{i_1, \cdots, i_j\}$ for $1 \leq j < M$, then we can write

$$\begin{aligned}
f(\bar{\mathbf{x}}) &\overset{(e)}{=} \sum_{j=1}^{M-1} F(\mathcal{T}_j)(\bar{x}_{i_j} - \bar{x}_{i_{j+1}}) + F(\bar{\mathcal{V}})\bar{x}_{i_M} \\
&\overset{(f)}{=} \sum_{j=1}^{M-1} \left[G(\mathcal{S} \cup \mathcal{T}_j) - G(\mathcal{S})\right](\bar{x}_{i_j} - \bar{x}_{i_{j+1}}) + \left[G(\mathcal{S} \cup \bar{\mathcal{V}}) - G(\mathcal{S})\right]\bar{x}_{i_M} \\
&= -G(\mathcal{S})\bar{x}_{i_1} + \sum_{j=1}^{M-1} G(\mathcal{S} \cup \mathcal{T}_j)(\bar{x}_{i_j} - \bar{x}_{i_{j+1}}) + G(\mathcal{S} \cup \bar{\mathcal{V}})\bar{x}_{i_M} \\
&= G(\mathcal{S})(1 - \bar{x}_{i_1}) + \sum_{j=1}^{M-1} G(\mathcal{S} \cup \mathcal{T}_j)(\bar{x}_{i_j} - \bar{x}_{i_{j+1}}) + G(\mathcal{S} \cup \bar{\mathcal{V}})(\bar{x}_{i_M} - 0) - G(\mathcal{S}) \\
&\overset{(g)}{=} g(\mathbf{1}_{\mathcal{S}}, \bar{\mathbf{x}}) - G(\mathcal{S})
\end{aligned}$$

where (e) and (g) were obtained according to the definition of the Lovász extension, and (f) followed (30), thus (31) is proved.

B PROOF OF THEOREM 1

We will prove the equivalence between (8) and (10).

Prove (8) $\rightarrow$ (10): The proof follows Lemmas 2 and 4.

Prove (10) $\rightarrow$ (8): We define a set function $F : 2^{\bar{\mathcal{V}}} \rightarrow \mathbb{R}$ as

$$F(\mathcal{T}) = \text{cut}_{\mathcal{G}}(\mathcal{S} \cup \mathcal{T}) - \text{cut}_{\mathcal{G}}(\mathcal{S}) \tag{32}$$

for any $\mathcal{T} \subseteq \bar{\mathcal{V}}$. According to Lemma 5, the function F is submodular and its Lovász extension f is such that for all $\bar{\mathbf{x}} \in [0,1]^M$,

$$f(\bar{\mathbf{x}}) = Q_1^{(g)}(\mathbf{1}_{\mathcal{S}}, \bar{\mathbf{x}}) - \text{cut}_{\mathcal{G}}(\mathcal{S}). \tag{33}$$

We can write

$$\begin{aligned}
\min_{\mathcal{T} \subseteq \bar{\mathcal{V}}} \text{cut}_{\mathcal{G}}(\mathcal{S} \cup \mathcal{T}) &\overset{(a)}{=} \min_{\mathcal{T} \subseteq \bar{\mathcal{V}}} F(\mathcal{T}) + \text{cut}_{\mathcal{G}}(\mathcal{S}) \\
&\overset{(b)}{=} \min_{\bar{\mathbf{x}} \in [0,1]^M} f(\bar{\mathbf{x}}) + \text{cut}_{\mathcal{G}}(\mathcal{S}) \\
&\overset{(c)}{=} \min_{\bar{\mathbf{x}} \in [0,1]^M} Q_1^{(g)}(\mathbf{1}_{\mathcal{S}}, \bar{\mathbf{x}}) \\
&\overset{(d)}{=} \min_{\bar{\mathbf{x}} \in \mathbb{R}^M} Q_1^{(g)}(\mathbf{1}_{\mathcal{S}}, \bar{\mathbf{x}}) \\
&\overset{(e)}{=} Q_1(\mathbf{1}_{\mathcal{S}}) = \text{cut}_{\mathcal{H}}(\mathcal{S})
\end{aligned}$$

where (a) followed (32), (b) followed (24), (c) followed (33), (d) followed Lemma 3, and (e) followed (10), thus (8) is obtained.

C PROOF OF THEOREM 2

Notice that the choice of the norm in the inner problem of Algorithm 2 only influences the scale of the solution. If we select the infinity norm, then we have

$$\begin{aligned}
&\min_{\|\mathbf{x}\|_\infty \le 1} Q_1(\mathbf{x}) - \lambda \langle \mathbf{x}, \boldsymbol{g} \rangle \\
&\overset{(a)}{=} \min_{\|\mathbf{x}\|_\infty \le 1} \min_{\bar{\mathbf{x}} \in \mathbb{R}^M} Q_1^{(g)}(\mathbf{x}, \bar{\mathbf{x}}) - \lambda \langle \mathbf{x}, \boldsymbol{g} \rangle \\
&= \min_{\bar{\mathbf{x}} \in \mathbb{R}^M} \min_{\|\mathbf{x}\|_\infty \le 1} Q_1^{(g)}(\mathbf{x}, \bar{\mathbf{x}}) - \lambda \langle \mathbf{x}, \boldsymbol{g} \rangle \\
&\overset{(b)}{=} \min_{\|\mathbf{y}\|_\infty \le 1} Q_1^{(g)}(\mathbf{y}) - \langle \mathbf{y}, \tilde{\boldsymbol{g}} \rangle
\end{aligned}$$

where (a) followed (10) and (b) followed Lemma 3 where we rewrite $Q_1^{(g)}(\mathbf{y}) = Q_1^{(g)}(\mathbf{x}, \bar{\mathbf{x}})$ for $\mathbf{y} = [\mathbf{x}^\top, \bar{\mathbf{x}}^\top]^\top$ and $\tilde{\boldsymbol{g}} = [\lambda \boldsymbol{g}^\top, \mathbf{0}_{1\times M}]^\top$. Then we replace the infinity norm with the Euclidean norm which only influences the scale of the solution.

D PROOF OF THEOREM 3

According to the Lovász extension of the cut function of a digraph, $Q_1^{(g)}(\mathbf{y})$ can be rewritten as

$$\begin{aligned}
Q_1^{(g)}(\mathbf{y}) &= \sum\nolimits_{u,v\in\mathcal{V}_{\mathcal{G}}} A_{uv}\max\{y_u - y_v, 0\} \\
&= \max_{\substack{\boldsymbol{\alpha}\in[0,1]^m \\ \alpha_{uv}+\alpha_{vu}=1}} \sum\nolimits_{(u,v)\in\tilde{\mathcal{E}}_{\mathcal{G}}} A_{uv}(y_u - y_v)\alpha_{uv} \\
&= \max_{\substack{\boldsymbol{\alpha}\in[0,1]^m \\ \alpha_{uv}+\alpha_{vu}=1}} \sum\nolimits_{(u,v)\in\tilde{\mathcal{E}}_{\mathcal{G}}} (A_{uv}\alpha_{uv} - A_{vu}\alpha_{vu})y_u \\
&= \max_{\substack{\boldsymbol{\alpha}\in[0,1]^m \\ \alpha_{uv}+\alpha_{vu}=1}} \sum\nolimits_{u\in\mathcal{V}_{\mathcal{G}}} y_u\left(\sum\nolimits_{v\,|\,(u,v)\in\tilde{\mathcal{E}}_{\mathcal{G}}} A_{uv}\alpha_{uv} - A_{vu}\alpha_{vu}\right) \\
&= \max_{\substack{\boldsymbol{\alpha}\in[0,1]^m \\ \alpha_{uv}+\alpha_{vu}=1}} \langle \mathbf{y}, f_A(\boldsymbol{\alpha})\rangle
\end{aligned}$$

It follows that

$$\begin{aligned}
&\min_{\|\mathbf{y}\|_2\le 1} Q_1^{(g)}(\mathbf{y}) - \langle \mathbf{y}, \tilde{\boldsymbol{g}}\rangle \\
&= \min_{\|\mathbf{y}\|_2\le 1} \max_{\substack{\boldsymbol{\alpha}\in[0,1]^m \\ \alpha_{uv}+\alpha_{vu}=1}} \langle \mathbf{y}, f_A(\boldsymbol{\alpha})\rangle - \langle \mathbf{y}, \tilde{\boldsymbol{g}}\rangle \\
&\overset{(a)}{=} \max_{\substack{\boldsymbol{\alpha}\in[0,1]^m \\ \alpha_{uv}+\alpha_{vu}=1}} \min_{\|\mathbf{y}\|_2\le 1} \langle \mathbf{y}, f_A(\boldsymbol{\alpha})\rangle - \langle \mathbf{y}, \tilde{\boldsymbol{g}}\rangle \\
&= \max_{\substack{\boldsymbol{\alpha}\in[0,1]^m \\ \alpha_{uv}+\alpha_{vu}=1}} \min_{\|\mathbf{y}\|_2\le 1} \langle \mathbf{y}, f_A(\boldsymbol{\alpha}) - \tilde{\boldsymbol{g}}\rangle \\
&\overset{(b)}{=} \max_{\substack{\boldsymbol{\alpha}\in[0,1]^m \\ \alpha_{uv}+\alpha_{vu}=1}} -\|f_A(\boldsymbol{\alpha}) - \tilde{\boldsymbol{g}}\|_2
\end{aligned}$$

where (a) followed Corollary 37.3.2 in (Rockafellar, 1970), and in (b) we used the solution to the minimization of the linear function over the Euclidean unit ball given by (14). Hence, the dual problem (13) is derived.

We rewrite the objective function $\Psi(\boldsymbol{\alpha})$ of the dual problem as

$$\Psi(\boldsymbol{\alpha}) = \sum\nolimits_{u\in\mathcal{V}_{\mathcal{G}}}\left(\left(\sum\nolimits_{v\,|\,(u,v)\in\tilde{\mathcal{E}}_{\mathcal{G}}} A_{uv}\alpha_{uv} - A_{vu}\alpha_{vu}\right) - \tilde{g}_u\right)^2.$$

It can be observed that the terms in $\Psi(\boldsymbol{\alpha})$ involving a specific α_{ij} are

$$\begin{aligned}
&\left(\left(\sum\nolimits_{v\,|\,(i,v)\in\tilde{\mathcal{E}}_{\mathcal{G}}} A_{iv}\alpha_{iv} - A_{vi}\alpha_{vi}\right) - \tilde{g}_i\right)^2 + \left(\left(\sum\nolimits_{v\,|\,(j,v)\in\tilde{\mathcal{E}}_{\mathcal{G}}} A_{jv}\alpha_{jv} - A_{vj}\alpha_{vj}\right) - \tilde{g}_j\right)^2 \\
&= \left(\left(\sum\nolimits_{v\,|\,(i,v)\in\tilde{\mathcal{E}}_{\mathcal{G}}} (A_{iv} + A_{vi})\alpha_{iv} - A_{vi}\right) - \tilde{g}_i\right)^2 + \left(\left(\sum\nolimits_{v\,|\,(j,v)\in\tilde{\mathcal{E}}_{\mathcal{G}}} A_{jv} - (A_{jv} + A_{vj})\alpha_{vj}\right) - \tilde{g}_j\right)^2
\end{aligned}$$

where we replaced α_{vi} and α_{jv} with $1-\alpha_{iv}$ and $1-\alpha_{vj}$, respectively. It follows that

$$\frac{\partial\Psi(\boldsymbol{\alpha})}{\partial\alpha_{ij}} = 2(A_{ij}+A_{ji})\left(\left(\sum\nolimits_{v\,|\,(i,v)\in\tilde{\mathcal{E}}_{\mathcal{G}}}(A_{iv}+A_{vi})\alpha_{iv}-A_{vi}\right)-\tilde{g}_i+\left(\sum\nolimits_{v\,|\,(j,v)\in\tilde{\mathcal{E}}_{\mathcal{G}}}(A_{jv}+A_{vj})\alpha_{vj}-A_{jv}\right)+\tilde{g}_j\right).$$

Thus, we have

$$\frac{\partial\Psi(\boldsymbol{\alpha})}{\partial\alpha_{ij}}-\frac{\partial\Psi(\boldsymbol{\beta})}{\partial\beta_{ij}} = 2(A_{ij}+A_{ji})\left(\sum\nolimits_{v\,|\,(i,v)\in\tilde{\mathcal{E}}_{\mathcal{G}}}(A_{iv}+A_{vi})(\alpha_{iv}-\beta_{iv})+\sum\nolimits_{v\,|\,(j,v)\in\tilde{\mathcal{E}}_{\mathcal{G}}}(A_{jv}+A_{vj})(\alpha_{vj}-\beta_{vj})\right).$$

It follows that

$$\begin{aligned}
\|\nabla\Psi(\boldsymbol{\alpha})-\nabla\Psi(\boldsymbol{\beta})\|_2^2 &= \sum_{\substack{(i,j)\in\tilde{\mathcal{E}}_{\mathcal{G}}\\ i<j}}\left(\frac{\partial\Psi(\boldsymbol{\alpha})}{\partial\alpha_{ij}}-\frac{\partial\Psi(\boldsymbol{\beta})}{\partial\beta_{ij}}\right)^2 \\
&= 4\sum_{\substack{(i,j)\in\tilde{\mathcal{E}}_{\mathcal{G}}\\ i<j}}(A_{ij}+A_{ji})^2\left(\sum_{v\,|\,(i,v)\in\tilde{\mathcal{E}}_{\mathcal{G}}}(A_{iv}+A_{vi})(\alpha_{iv}-\beta_{iv})+\sum_{v\,|\,(j,v)\in\tilde{\mathcal{E}}_{\mathcal{G}}}(A_{jv}+A_{vj})(\alpha_{vj}-\beta_{vj})\right)^2 \\
&\overset{(a)}{\leq} 8\sum_{\substack{(i,j)\in\tilde{\mathcal{E}}_{\mathcal{G}}\\ i<j}}(A_{ij}+A_{ji})^2\left(\left(\sum_{v\,|\,(i,v)\in\tilde{\mathcal{E}}_{\mathcal{G}}}(A_{iv}+A_{vi})(\alpha_{iv}-\beta_{iv})\right)^2+\left(\sum_{v\,|\,(j,v)\in\tilde{\mathcal{E}}_{\mathcal{G}}}(A_{jv}+A_{vj})(\alpha_{vj}-\beta_{vj})\right)^2\right) \\
&\overset{(b)}{\leq} 8\sum_{\substack{(i,j)\in\tilde{\mathcal{E}}_{\mathcal{G}}\\ i<j}}(A_{ij}+A_{ji})^2\left(\sum_{v\,|\,(i,v)\in\tilde{\mathcal{E}}_{\mathcal{G}}}(A_{iv}+A_{vi})^2\sum_{v\,|\,(i,v)\in\tilde{\mathcal{E}}_{\mathcal{G}}}(\alpha_{iv}-\beta_{iv})^2+\sum_{v\,|\,(j,v)\in\tilde{\mathcal{E}}_{\mathcal{G}}}(A_{jv}+A_{vj})^2\sum_{v\,|\,(j,v)\in\tilde{\mathcal{E}}_{\mathcal{G}}}(\alpha_{jv}-\beta_{jv})^2\right) \\
&= 8\sum_{(i,j)\in\tilde{\mathcal{E}}_{\mathcal{G}}}(A_{ij}+A_{ji})^2\left(\sum_{v\,|\,(i,v)\in\tilde{\mathcal{E}}_{\mathcal{G}}}(A_{iv}+A_{vi})^2\sum_{v\,|\,(i,v)\in\tilde{\mathcal{E}}_{\mathcal{G}}}(\alpha_{iv}-\beta_{iv})^2\right) \\
&= 8\sum_{i\in\mathcal{V}_{\mathcal{G}}}\left(\sum_{v\,|\,(i,v)\in\tilde{\mathcal{E}}_{\mathcal{G}}}(A_{iv}+A_{vi})^2\right)^2\sum_{v\,|\,(i,v)\in\tilde{\mathcal{E}}_{\mathcal{G}}}(\alpha_{iv}-\beta_{iv})^2 \\
&\leq 8\left(\max_{i\in\mathcal{V}_{\mathcal{G}}}\sum_{v\,|\,(i,v)\in\tilde{\mathcal{E}}_{\mathcal{G}}}(A_{iv}+A_{vi})^2\right)^2\sum_{(i,v)\in\tilde{\mathcal{E}}_{\mathcal{G}}}(\alpha_{iv}-\beta_{iv})^2 \\
&= 16\left(\max_{i\in\mathcal{V}_{\mathcal{G}}}\sum_{v\,|\,(i,v)\in\tilde{\mathcal{E}}_{\mathcal{G}}}(A_{iv}+A_{vi})^2\right)^2\|\boldsymbol{\alpha}-\boldsymbol{\beta}\|_2^2
\end{aligned}$$

where (a) leveraged the inequality $(a+b)^2\leq 2(a^2+b^2)$ and (b) followed the Cauchy-Schwarz inequality. An upper bound on the Lipschitz constant of $\nabla\Psi$ is thus obtained.

REFERENCES

Amghibech, S. (2003). Eigenvalues of the discrete p-laplacian for graphs. *Ars Combinatoria* 67, 283–302

Bach, F. (2013). Learning with submodular functions: A convex optimization perspective. *Foundations and Trends® in Machine Learning* 6, 145–373. https://doi.org/10.1561/2200000039

Beck, A. and Teboulle, M. (2009). A fast iterative shrinkage-thresholding algorithm for linear inverse problems. *SIAM Journal on Imaging Sciences* 2, 183–202. https://doi.org/10.1137/080716542

Benson, A. R., Gleich, D. F., and Leskovec, J. (2016). Higher-order organization of complex networks. *Science* 353, 163–166. https://doi.org/10.1126/science.aad9029

Boyd, S., Boyd, S. P., and Vandenberghe, L. (2004). *Convex optimization* (Cambridge university press)

Bühler, T. and Hein, M. (2009). Spectral clustering based on the graph p-laplacian. In *International Conference on Machine Learning*. 81–88. https://doi.org/10.1145/1553374.1553385

Chambolle, A. and Pock, T. (2011). A first-order primal-dual algorithm for convex problems with applications to imaging. *Journal of mathematical imaging and vision* 40, 120–145. https://doi.org/10.1007/s10851-010-0251-1

Chambolle, A. and Pock, T. (2016a). An introduction to continuous optimization for imaging. *Acta Numerica* 25, 161–319. https://doi.org/10.1017/S096249291600009X

Chambolle, A. and Pock, T. (2016b). On the ergodic convergence rates of a first-order primal–dual algorithm. *Mathematical Programming* 159, 253–287. https://doi.org/10.1007/s10107-015-0957-3

Chang, K.-C. (2016). Spectrum of the 1-laplacian and cheeger's constant on graphs. *Journal of Graph Theory* 81, 167–207. https://doi.org/10.1002/jgt.21871

Chang, K.-C., Shao, S., and Zhang, D. (2017). Nodal domains of eigenvectors for 1-laplacian on graphs. *Advances in Mathematics* 308, 529–574. https://doi.org/10.1016/j.aim.2016.12.020

Chitra, U. and Raphael, B. (2019). Random walks on hypergraphs with edge-dependent vertex weights. In *International Conference on Machine Learning*. 1172–1181. http://proceedings.mlr.press/v97/chitra19a.html

Ene, A. and Nguyen, H. (2015). Random coordinate descent methods for minimizing decomposable submodular functions. In *International Conference on Machine Learning*. 787–795. http://proceedings.mlr.press/v37/ene15.html

Fu, G., Zhao, P., and Bian, Y. (2022). p-laplacian based graph neural networks. In *International Conference on Machine Learning*. 6878–6917. https://proceedings.mlr.press/v162/fu22e.html

Hayashi, K., Aksoy, S. G., Park, C. H., and Park, H. (2020). Hypergraph random walks, laplacians, and clustering. In *Conference on Information and Knowledge Management*. 495–504. https://doi.org/10.1145/3340531.3412034

Hein, M. and Bühler, T. (2010). An inverse power method for nonlinear eigenproblems with applications in 1-spectral clustering and sparse PCA. *Advances in Neural Information Processing Systems* 23. https://dl.acm.org/doi/10.5555/2997189.2997284

Hein, M. and Setzer, S. (2011). Beyond spectral clustering - tight relaxations of balanced graph cuts. *Advances in neural information processing systems* 24. https://dl.acm.org/doi/10.5555/2986459.2986723

Hein, M., Setzer, S., Jost, L., and Rangapuram, S. S. (2013). The total variation on hypergraphs-learning on hypergraphs revisited. *Advances in Neural Information Processing Systems* 26. https://dl.acm.org/doi/10.5555/2999792.2999883

Jegelka, S., Bach, F., and Sra, S. (2013). Reflection methods for user-friendly submodular optimization. *Advances in Neural Information Processing Systems* 26. https://dl.acm.org/doi/10.5555/2999611.2999758

Leskovec, J., Rajaraman, A., and Ullman, J. D. (2020). *Mining of massive data sets*. https://doi.org/10.1017/CBO9781139924801

Lewis, D. D., Yang, Y., Russell-Rose, T., and Li, F. (2004). RCV1: A new benchmark collection for text categorization research. *Journal of Machine Learning Research* 5, 361–397. https://dl.acm.org/doi/10.5555/1005332.1005345

Li, J., He, J., and Zhu, Y. (2018). E-tail product return prediction via hypergraph-based local graph cut. In *International Conference on Knowledge Discovery & Data Mining*. 519–527. https://doi.org/10.1145/3219819.3219829

Li, P. and Milenkovic, O. (2017). Inhomogeneous hypergraph clustering with applications. *Advances in Neural Information Processing Systems* 30. https://dl.acm.org/doi/10.5555/3294771.3294991

Li, P. and Milenkovic, O. (2018). Submodular hypergraphs: p-laplacians, cheeger inequalities and spectral clustering. In *International Conference on Machine Learning*. 3014–3023. http://proceedings.mlr.press/v80/li18e.html

Liu, M., Veldt, N., Song, H., Li, P., and Gleich, D. F. (2021). Strongly local hypergraph diffusions for clustering and semi-supervised learning. In *International World Wide Web Conference*. 2092–2103. https://doi.org/10.1145/3442381.3449887

Lovász, L. (1983). Submodular functions and convexity. In *Mathematical programming The state of the art*. 235–257. https://doi.org/10.1007/978-3-642-68874-4_10

Nesterov, Y. E. (1983). A method for solving the convex programming problem with convergence rate o (1/kˆ 2). In *Dokl. akad. nauk Sssr*. vol. 269, 543–547

Rockafellar, R. T. (1970). *Convex analysis*, vol. 18. https://doi.org/10.1515/9781400873173

Schaub, M. T., Zhu, Y., Seby, J.-B., Roddenberry, T. M., and Segarra, S. (2021). Signal processing on higher-order networks: Livin'on the edge... and beyond. *Signal Processing* 187, 108149. https://doi.org/10.1016/j.sigpro.2021.108149

Szlam, A. and Bresson, X. (2010). Total variation and cheeger cuts. In *International Conference on Machine Learning*. 1039–1046. https://dl.acm.org/doi/10.5555/3104322.3104454

Tudisco, F. and Hein, M. (2018). A nodal domain theorem and a higher-order cheeger inequality for the graph p-laplacian. *Journal of Spectral Theory* 8, 883–908. https://doi.org/10.4171/JST/216

Tudisco, F., Mercado, P., and Hein, M. (2018). Community detection in networks via nonlinear modularity eigenvectors. *SIAM Journal on Applied Mathematics* 78, 2393–2419. https://doi.org/10.1137/17M1144143

Veldt, N., Benson, A. R., and Kleinberg, J. (2020). Hypergraph cuts with general splitting functions. *arXiv preprint arXiv:2001.02817* https://arxiv.org/abs/2001.02817

Von Luxburg, U. (2007). A tutorial on spectral clustering. *Statistics and Computing* 17, 395–416. https://doi.org/10.1007/s11222-007-9033-z

Wagner, D. and Wagner, F. (1993). Between min cut and graph bisection. In *International Symposium on Mathematical Foundations of Computer Science*. 744–750. https://doi.org/10.1007/3-540-57182-5_65

Yoshida, Y. (2019). Cheeger inequalities for submodular transformations. In *ACM-SIAM Symposium on Discrete Algorithms*. 2582–2601. https://doi.org/10.1137/1.9781611975482.160

Zhu, Y., Li, B., and Segarra, S. (2021). Co-clustering vertices and hyperedges via spectral hypergraph partitioning. In *European Signal Processing Conference*. 1416–1420. https://doi.org/10.23919/EUSIPCO54536.2021.9616223

Zhu, Y., Li, B., and Segarra, S. (2022). Hypergraphs with edge-dependent vertex weights: Spectral clustering based on the 1-laplacian. In *International Conference on Acoustics, Speech and Signal Processing*. 8837–8841. https://doi.org/10.1109/ICASSP43922.2022.9746363

Zhu, Y. and Segarra, S. (2022). Hypergraph cuts with edge-dependent vertex weights. *Applied Network Science* 7, 45. https://doi.org/10.1007/s41109-022-00483-x
